# The effect of word sense disambiguation accuracy on literature based discovery

**DOI:** 10.1186/s12911-016-0296-1

**Published:** 2016-07-18

**Authors:** Judita Preiss, Mark Stevenson

**Affiliations:** Advanced Computing Research Center, Department of Computer Science, The University of Sheffield, 211 Portobello, Sheffield, S1 4DP UK

**Keywords:** Data mining, Text processing, Literature based discovery, Word sense disambiguation

## Abstract

**Background:**

The volume of research published in the biomedical domain has increasingly lead to researchers focussing on specific areas of interest and connections between findings being missed. Literature based discovery (LBD) attempts to address this problem by searching for previously unnoticed connections between published information (also known as “hidden knowledge”). A common approach is to identify hidden knowledge via shared linking terms. However, biomedical documents are highly ambiguous which can lead LBD systems to over generate hidden knowledge by hypothesising connections through different meanings of linking terms. Word Sense Disambiguation (WSD) aims to resolve ambiguities in text by identifying the meaning of ambiguous terms. This study explores the effect of WSD accuracy on LBD performance.

**Methods:**

An existing LBD system is employed and four approaches to WSD of biomedical documents integrated with it. The accuracy of each WSD approach is determined by comparing its output against a standard benchmark. Evaluation of the LBD output is carried out using timeslicing approach, where hidden knowledge is generated from articles published prior to a certain cutoff date and a gold standard extracted from publications after the cutoff date.

**Results:**

WSD accuracy varies depending on the approach used. The connection between the performance of the LBD and WSD systems are analysed to reveal a correlation between WSD accuracy and LBD performance.

**Conclusion:**

This study reveals that LBD performance is sensitive to WSD accuracy. It is therefore concluded that WSD has the potential to improve the output of LBD systems by reducing the amount of spurious hidden knowledge that is generated. It is also suggested that further improvements in WSD accuracy have the potential to improve LBD accuracy.

## Background

The rapid growth in the number of academic publications makes it increasingly difficult for researchers to keep up to date with advances in their areas of interest. In some fields, such as those related to medicine, the volume of published research is now so great that no individual can read all of the publications that are potentially relevant to their research. Consequently researchers focus on key publications within their own domain, but this can lead to connections between sub-fields being missed. Literature-based discovery (LBD) aims to (semi-)automate the process of identifying inferable connections. Swanson [1] proposed the A-B-C model for finding links between two unconnected terms, *A* and *C*. The approach operates by identifying a publication containing both *A* and *B* and another containing *B* and *C* (for some linking term *B*). The approach’s efficacy was demonstrated by finding a link between *Raynaud disease* and *fish oil* via *blood viscosity*.

Two types of LBD have been discussed in the literature: open and closed [2]. Open discovery starts from term *A*, follows connections to any *B* terms which are further followed to any *C* terms. Removing directly related *A*−*C* pairs from the list leaves hypothesized new knowledge. In closed discovery, both the *A* and *C* terms are specified at the start and only the *B* terms are sought. The *B* terms can provide justification for any hypothesized link between *A* and *C*.

For both open and closed discovery, identifying relations between pairs of terms is obviously critical to the success of an LBD system. A simple approach is to assume that terms which appear together (e.g. occur in the same document title, sentence or document) are related. However, this assumption causes a large amount of over-generation since connections are hypothesised through linking terms which are unrelated or too general.

We note the following types of linking terms which tend to over-generate connections:

1. Non-content words (words such as *and* and *or*).
2. Uninformative or very general words (such as *patient* or *week*).
3. Ambiguous terms (words with multiple meanings such as *cold* which can mean *common cold*, *cold sensation* or *Chronic Obstructive Lung Disease (COLD)*).

Non-content words (point 1) can be addressed using a stoplist. Uninformative words (point 2) are more difficult to identify than content words: they often appear in inventories, but do not provide much information for the task. For example, *patient* appears in the UMLS Metathesaurus [3], but it is rarely an informative term for LBD. A list of uninformative words can be built either automatically (with varying degrees of human intervention) or fully manually; e.g. Swanson, Smalheiser, and Torvik [4] build (semi-automatically) a 9,500 word stoplist for their LBD system. Such a list often suffers from errors of omission, and in this case, the list has been criticized for being too fine tuned to a fundamental LBD discovery [5]. Another approach to removing uninformative words carries this out at the system level, either by using an LBD system to indicate commonly occurring (and thus likely uninformative) linking terms (building a stoplist), or by removing links where these are likely to be unhelpful [6].

Point 3 is the central focus of this work. Ambiguous terms can lead to spurious hidden knowledge being identified: if a publication contains a connection between *A* and *B*_1_ and another supports a connection between *B*_2_ and *C* (where *B*_1_ and *B*_2_ are different senses of the term *B*) the A-B-C model will suggest a hidden connection between *A* and *C*, despite there being no link.

The problem is exacerbated by the prevalence of ambiguous terms in the biomedical literature. A range of different types can be found [7] including:

1. **ambiguous words**, e.g. *depression* can refer to *psychological condition* or *hollow on surface* [8].
2. **abbreviations with multiple possible expansions** [9], e.g. *CAT* can mean *chloramphenicol acetyl transferase*, *computer-aided testing*, *computer-automated tomography*, *choline acetyltransferase* or *computed axial tomography* [10].
3. **gene names** are often not used uniquely and the same description can be used to refer to different genes [11], e.g. *NAP1* relates to at least five genes.

The standard approach to LBD of identifying connections between words fails to account for word ambiguity, and consequently some researchers have explored the use of alternative representations for words. Weeber et al [5] discuss the disadvantages of generating hidden knowledge from words, or *n*-grams of words, as opposed to generating from Concept Unique Identifiers (CUIs) from the UMLS Metathesaurus, although they only indirectly point out the sense disambiguating advantage by higlighting that any stop lists used for filtering terms no longer needs to be domain specific. They employ the publicly available tool MetaMap [12], which assigns a CUI to each term, and thus avoid ambiguous linking terms. However, they do not discuss the extent to which LBD is sensitive to the accuracy of the WSD system employed or whether performance gains are due to the filtering of irrelevant terms.

It seems plausible that the information provided by WSD will improve performance of language processing tasks such as LBD. However, WSD has proved to be a challenging problem and the errors made by WSD systems often mean that integrating them with language processing systems has not lead to performance increases in practise [13]. For example, it is unclear whether WSD benefits Machine Translation (MT), a key NLP application, with researchers making opposing claims about the effect on performance [14–16]. A similar situation is observed for Information Retrieval where it was thought that applying WSD did not improve performance [17], although more recent work has suggested that it can [18]. Consequently, it is not possible to predict whether WSD will be useful for any application, including LBD, a priori and its effect needs to be evaluated directly.

We explore the connection between WSD accuracy and the effectiveness of LBD. We examine the effect a number of WSD approaches with significantly different performance have on the hidden knowledge generated by an LBD system. LBD performance is evaluated using a time-slicing approach [19].

## Methods

### Literature based discovery system

We employ an LBD system based on the A-B-C model [1]. Title co-occurrence is used as the relation between concepts to allow comparison with previous work. A pair of CUIs, *A* and *B*, is considered to be related if both CUIs (as determined by the chosen WSD system) appear in the title of a Medline publication.

Our LBD system [20] builds a matrix *A* in which element *a*_*ij*_ describes the frequency with which a CUI, *c*_*i*_, is linked to (by title co-occurrence) another CUI, *c*_*j*_, in the document collection (all our experiments are performed on a document collection consisting of Medline abstracts published between 2000 and 2005). Non-zero elements of the matrix *A* indicate pairs of CUIs that are directly related. The square of this matrix, *A*^2^, indicates pairs of CUIs that are indirectly connected, via an intermediate one [21]. Consequently, non-zero elements of *A*^2^ that are zero in *A* are hidden knowledge. This approach can be used for open discovery – for any CUI the system will generate all terms that can be reached via any single linking term.

#### Advantages of using CUIs

Using UMLS CUIs rather than terms helps to avoid the generation of spurious hidden knowledge. Non content words are removed indirectly since the majority of these are not included in the set of UMLS CUIs. Uninformative or very general terms can also be removed by making use of the UMLS Semantic Network, which assigns one of 133 possible semantic types to each CUI. Many of these categories are obviously unhelpful for LBD (such as *geographical location*) and were removed: 70 semantic types were manually selected for removal by examining the terms associated with them and evaluating them for their potential use in LBD. Finally, the use of CUIs avoids the generation of spurious hidden knowledge due to lexical ambiguity since each CUI refers to a single meaning of an ambiguous term.

### Word sense disambiguation

We explore three different WSD systems for the biomedical domain, a general personalized page rank (PPR) based system [22] which we apply to the biomedical domain, a vector space model (VSM) based WSD system [23] applicable to any domain but tuned to biomedical texts, and MetaMap [12] which is designed to associate terms in biomedical documents with UMLS CUIs. We also present results based on a random sense baseline.

#### PPR WSD system

The PPR system builds a graph from a knowledge base and applies the personalized PageRank algorithm to rank the vertices and thus assign senses to each target word. It was applied to the biomedical domain by using information from the UMLS’s MRREL table to create a graph and found to outperform other WSD approaches based on information from the UMLS [24]. We employ PPR, available from http://ixa2.si.ehu.es/ukb/, in the *ppr_w2w* mode which assigns all senses in a context in a single pass (rather than applying a sliding window).

#### VSM based WSD system

The PPR system is unsupervised, i.e. does not make use of any annotated data. Supervised WSD systems, which make use of annotated data, generally perform better than unsupervised approaches but can only disambiguate those words for which annotations exist.

The DALE [25] system makes use of data annotated automatically (using the monosemous relatives and co-occuring concepts approaches [7]). Word instances are converted to a feature vector based on the lemmas of all content words within the same sentence as well as the lemmas of all words in a window of ±4 words. A Vector Space Model (VSM) is used to compare the vector representing an ambiguous word against the centroid vectors of each candidate CUI and the most similar chosen.

#### MetaMap

The MetaMap WSD system [12], available from http://metamap.nlm.nih.gov/, maps terms to their UMLS CUI represenation using rules and patterns.

#### Random baseline

To allow comparison with a less accurate WSD system, LBD is also generated from a random sense assignment. In this case, one of the possible UMLS CUIs is selected at random for each word. Note that it is not possible to produce a comparable result for the system when no WSD is employed. This is because a CUI based gold standard cannot trivially be mapped to a term based gold standard. CUI descriptions are usually very specific and unlikely to appear in text directly - for example, CUI C085292 corresponds to Raynaud’s phenomenon aggravated, which is unlikely to appear in a document, and thus most hidden knowledge generated directly from terms will likely not appear in such a gold standard. In addition, a a gold standard generated directly from terms will be of a different size to that used for evaluation of the WSD system (due to multiple senses of a term being represented by a single word).

## Results

To examine the effect of WSD on LBD, it is necessary to (a) directly evaluate the WSD systems, and (b) evaluate the LBD knowledge acquired when the various WSD systems are employed. A comparison of the WSD systems is presented first followed by evaluation of its effect on LBD.

### WSD performance

The MSH WSD test collection, available from http://wsd.nlm.nih.gov/, was used to evaluate the WSD systems. The collection contains instances of 203 ambiguous words and terms annotated with the relevant CUI from the 2009AA version of UMLS [26]. There are up to 100 instances of each ambiguous term.

Table 1 shows the precision, recall and *F*-measure for each system when their output is compared against the MSH WSD annotation. There are clear performance differences between the approaches. As MetaMap is the only algorithm attempting to identify multiword units, the lower recall (and also precision, as multiword units are likely to have fewer senses and therefore are easier to disambiguate) of the remaining WSD algorithms is likely due to this. With some algorithm tuning, it may be possible to use MetaMap as a pre-processor and thus boost performance of the non-MetaMap WSD systems.

**Table 1:** Performance of the WSD systems

WSD	Precision	Recall	F-measure
MetaMap	51.3 %	43.1 %	46.8 %
VSM	46.7 %	24.8 %	32.4 %
PPR	40.1 %	23.3 %	29.5 %
Random	29.3 %	29.3 %	29.3 %

### WSD and LBD performance

Evaluating LBD is obviously not an easy task as no gold standard can be constructed for hidden knowledge. Two main evaluation techniques exist: replication of existing discoveries and timeslice evaluation. The replication approach involves using a new LBD system to reproduce a previously verified discovery. However, only a small number of hidden knowledge discoveries has been published (we have found fewer than 10 published hidden knowledge discoveries), and with a small evaluation set, one missed connection (which can be due to a simple misidentification of a multiword) is very noticeable.

The second approach is timeslicing [19], where hidden knowledge is generated from articles published prior to a certain cutoff date and a gold standard is extracted from publications after the cutoff date (any ‘knowledge’ appearing in publications after the cutoff is deemed new). The hidden knowledge is evaluated against the gold standard, allowing many more hidden knowledge pairs to be compared than in replication. For our evaluation, the gold standard is generated by extracting title co-occurrence pairs from the segment after the cutoff date (2006 onwards) and eliminating any title co-occurrence pairs appearing from the start of Medline up to the cutoff date (1700-2005): this results in 2,320,301 pairs of ‘new published knowledge’ from the 6,858,042 abstracts in this time range.

The results of LBD based on title co-occurrence performed on the 2000-2005 segment of Medline combined with the five sense assignment techniques are presented in Table 2. Given the quantity of hidden knowledge generated, the F-measure will show strong bias towards systems which output fewer hidden links; we therefore present F-measure scaled by the number of links generated (normalized so that the highest performing system, MetaMap, is scaled to 1 – i.e. all F-measures are divided by MetaMap’s F-measure).

**Table 2:** Performance of the LBD system

WSD	# pairs	Scaled F-measure
MetaMap	4,554,466,783	1.000
VSM	175,748,768	0.038
PPR	162,065,341	0.035
Random	133,004,828	0.029

## Discussion

The results show that WSD system performance has a clear impact on the results obtained from the LBD system using time slicing. The highest F-measure is obtained using the best WSD approach (in this case, MetaMap). Performance drops with the decreasing F-measure of the WSD algorithm used and the results therefore suggest a direct connection between WSD and LDB performance. Accuracy is particularly important for LBD given the amount of hidden knowledge which is generated, since LBD systems have the potential to generate more candidates for hidden knowledge than can reasonably be explored. For example, our LBD system generates over 4.5 billion hidden knowledge candidates when MetaMap is used to carry out WSD.

## Conclusions

This paper explores how the problem of lexical ambiguity affects the performance of LBD systems and the extent to which WSD could be applied to solve this issue. WSD approaches with varying levels of accuracy were combined with an LBD system based on the A-B-C model. Evaluation of the hidden knowledge generated was carried out using the time-slicing approach and revealed that LBD is sensitive to the accuracy of the underlying WSD system. We therefore conclude that WSD forms a useful component of LBD systems and suggest that further improvements in WSD accuracy could benefit LBD.

For future work, we would like to carry out further experiments using other WSD systems. In addition we would also like to make use of LBD systems which use wider sources of information to identify the relations between concepts mentioned in documents.
